# Modeling and Simulation of Linear and Nonlinear MEMS Scale Electromagnetic Energy Harvesters for Random Vibration Environments

**DOI:** 10.1155/2014/742580

**Published:** 2014-01-30

**Authors:** Farid Khan, Boris Stoeber, Farrokh Sassani

**Affiliations:** ^1^Department of Mechanical Engineering, The University of British Columbia, Vancouver, BC, Canada V6T 1Z4; ^2^Institute of Mechatronics Engineering, University of Engineering and Technology, Peshawar, Pakistan; ^3^Department of Electrical and Computer Engineering, The University of British Columbia, Vancouver, BC, Canada V6T 1Z4

## Abstract

The simulation results for electromagnetic energy harvesters (EMEHs) under broad band stationary Gaussian random excitations indicate the importance of both a high transformation factor and a high mechanical quality factor to achieve favourable mean power, mean square load voltage, and output spectral density. The optimum load is different for random vibrations and for sinusoidal vibration. Reducing the total damping ratio under band-limited random excitation yields a higher mean square load voltage. Reduced bandwidth resulting from decreased mechanical damping can be compensated by increasing the electrical damping (transformation factor) leading to a higher mean square load voltage and power. Nonlinear EMEHs with a Duffing spring and with linear plus cubic damping are modeled using the method of statistical linearization. These nonlinear EMEHs exhibit approximately linear behaviour under low levels of broadband stationary Gaussian random vibration; however, at higher levels of such excitation the central (resonant) frequency of the spectral density of the output voltage shifts due to the increased nonlinear stiffness and the bandwidth broadens slightly. Nonlinear EMEHs exhibit lower maximum output voltage and central frequency of the spectral density with nonlinear damping compared to linear damping. Stronger nonlinear damping yields broader bandwidths at stable resonant frequency.

## 1. Introduction

The growing demand for autonomous and self-powered sensors [1] has resulted in immense interest in harvesting energy from the environment. Similar to other energy harvesting techniques (solar, acoustic, thermal, or wind) [1, 2], harvesting energy from ambient mechanical vibrations [3] with piezoelectric [4], electrostatic [5], and electromagnetic [6] energy harvesters has gained increasing interest in recent years. Mechanical vibrations are abundant in the environment in the form of machine vibration [7] and the vibration of household and office appliances [8]. These sources have sufficient vibration levels to generate power to run ultralow power (ULP) sensors [9] and ULP electronic circuitry; however, the frequency content of these vibrations is spread over a wide range.

Most of the developed linear and nonlinear resonant energy harvesters have been tested and characterized under harmonic excitations; however, real environmental vibrations do not contain one steady single frequency but the vibration is rather distributed over a broad band of frequencies and is random in nature. The power spectral density (PSD) of the acceleration along the tangential direction of a car tire at a speed of 50 km/h, for example, has a rich energy content in a broad band from 5 Hz to 1 kHz [10]. The vibration of a car driven on a highway at about 105 km/h ranges from 1 to 500 Hz [11]. The vibration levels of household appliances reported in [12] also cover a broad bandfrom 1 to 500 Hz.

The models developed to predict the performance of microfabricated linear resonant energy harvesters under harmonic excitation [13–19] are not suitable to estimate the performance of the same devices when subjected to a narrow or a broad band of random vibration. A model for piezoelectric energy harvesters under broad band random vibration has been developed by Adhikari et al. in [20], where they assume the ambient base excitation as a stationary Gaussian white noise with constant spectral density (SD) over the considered frequency range. A circuit simulator, the Simulation Program with Integrated Circuit Emphasis (SPICE), is used in [21, 22] to study an energy harvester under broad band random vibrations. With the same SPICE technique an electrostatic energy harvester has been simulated for input acceleration spectral densities of 5 × 10^−5^ and 5 × 10^−4^ g^2^/Hz [21]. A two-port transducer model developed for performance tracking of linear electromechanical energy harvesters under random broad band excitation [23] has been extended for the analysis of linear and nonlinear piezoelectric and electrostatic harvesters excited by broad band and narrow band random vibrations [24]. Simulations of the harvester output power, proof mass displacement, and optimum load have been performed under broad band Gaussian white noise and band limited noise excitation. The author [23] has suggested a mapping method to extend the model application to electromagnetic energy harvesters.

This paper presents the analytical modeling and simulation results for linear and nonlinear resonant electromagnetic energy harvesters (EMEHs) under broad band and narrow band excitations. The models are parameterized such that they are also applicable to other types of linear and nonlinear resonant EMEHs. Resonant EMEHs with moving magnet or moving coil architecture, with wound coil or planar coil, and with uniform magnetic field or nonuniform magnetic field configuration, all can be investigated. The results of this work can be utilized for design and performance estimation of MEMS scale linear and nonlinear EMEHs under random vibrations. For broad band and narrow band random excitations, spectral densities (SDs) of load voltage and load power, mean square load voltage and mean power delivered to the load can be predicted for the harvester design parameters, such as the mechanical quality factor, the transformation factor, and the natural frequency. Nonlinear harvesters with only spring nonlinearity and with both spring and damping nonlinearities have been modeled using the method of statistical linearization. These nonlinear models are useful in investigating the effects of mechanical nonlinearity on the performance and bandwidth of the harvesters, when they are subjected to random vibrations.

## 2. Modeling

**Figure 1:**
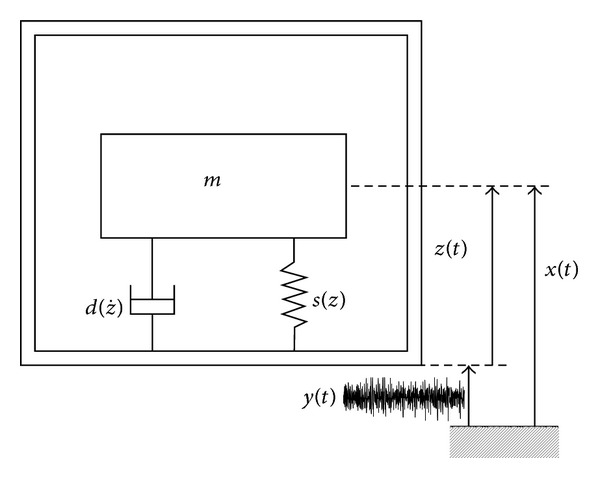
Lumped parameter model of an inertial electromagnetic energy harvester.

The EMEHs are seismic or inertial devices consisting of an inertial mass *m* being the magnet or a proof mass and a suspension with the restoring spring force *s*(*z*) to support the magnet or the coil. During operation the motion of the inertial mass is damped by a damping force $d(\dot{z})$ that arises due to mechanical damping (air, material, and support damping) and electrical damping induced when current flows in the coil. The linear and nonlinear EMEHs can be modeled as single degree of freedom, spring-mass-damper systems with base excitation as shown in Figure 1.

For an excitation $\ddot{y}(t)$, the general form of the equation of motion for an inertial EMEH

(1) $$\begin{array}{c} m\ddot{z}+\text{d}\left(\dot{z}\right)+s\left(z\right)=-m\ddot{y} \end{array}$$

depends on the relative acceleration $\ddot{z}(t)$, relative velocity $\dot{z}(t)$, and relative displacement *z*(*t*) between the permanent magnet and the coil. The expressions for the damping force $\text{d}(\dot{z})$ and the spring force *s*(*z*) are modeled according to the physical nature of the damping and stiffness present in the harvester. Depending on the architecture and design of the EMEH and the excitation conditions, both spring force and damping force can be linear or one or both can be nonlinear. The behaviour of a linear EMEH (both *s*(*z*) and $\text{d}(\dot{z})$ are linear) and a nonlinear EMEH (one or both *s*(*z*) and $\text{d}(\dot{z})$ are nonlinear) is different and requires separate models to investigate their performance under broad band or narrow band excitations.

### 2.1. Harvester with Linear Stiffness and Linear Damping

In linear EMEHs the spring force *s*(*z*) = *kz* and the damping force $d(\dot{z})=b_{T}\dot{z}$ are described by the linear spring stiffness *k* and the linear total damping coefficient *b*_*T*_ = *b*_*m*_ + *b*_*e*_, respectively. The mechanical damping coefficient *b*_*m*_ and the electrical damping coefficient *b*_*e*_ contribute to the total damping of the harvester. The equation of motion, (1), for linear EMEHs reduces to

(2) $$\begin{array}{c} m\ddot{z}+b_{T}\dot{z}+kz=-m\ddot{y} \end{array}$$

or

(3) $$\begin{array}{c} \ddot{z}+2\zeta_{T}\omega_{n}\dot{z}+\omega_{n}^{2}z=-\ddot{y}, \end{array}$$

expressed in terms of the natural frequency *ω*_*n*_ and the total damping ratio *ζ*_*T*_ of the system.

The complex frequency response of the system

(4) $$\begin{array}{c} H\left(i\omega \right)=\frac{\omega }{\left(\omega_{n}^{2}-\omega^{2}\right)i-2\zeta_{T}\omega_{n}\omega } \end{array}$$

is obtained using Fourier analysis by letting $\ddot{y}(t)=Ae^{i\omega t}$ and $\dot{z}(t)=Ue^{i\omega t}$ in (3).

The magnitude of the complex frequency response

(5) $$\begin{array}{c} \left|H\left(i\omega \right)\right|=\frac{\left(\omega /\omega_{n}\right)}{\omega_{n}\sqrt{{\left[1-{\left(\omega /\omega_{n}\right)}^{2}\right]}^{2}+{\left[2\zeta_{T}\left(\omega /\omega_{n}\right)\right]}^{2}}} \end{array}$$

and the SD of the base acceleration *S*_*A*_(*ω*) yield the SD of the relative velocity

(6) $$\begin{array}{c} S_{U}\left(\omega \right)={\left|H\left(i\omega \right)\right|}^{2}S_{A}\left(\omega \right). \end{array}$$

The open-circuit voltage induced in EMEHs [17]

(7) $$\begin{array}{c} V_{G}\left(t\right)=G\dot{z}\left(t\right) \end{array}$$

across the coil is directly proportional to the transformation factor *G*. The transformation factor *G* describes the coupling between the mechanical and electrical energy domains of the EMEH and greatly influences the energy conversion between these two domains. For EMEHs that have a uniform magnetic field perpendicular to the coil displacement [13], the transformation factor

(8) $$\begin{array}{c} G=BL \end{array}$$

results from the uniform magnetic flux density *B* and the effective length of the coil *L*.

In EMEHs with nonuniform magnetic field configuration [17], where the coil moves in the magnetic field direction, the transformation factor

(9) $$\begin{array}{c} G=S\frac{dB_{z}}{\text{d}z} \end{array}$$

depends on the gradient *dB*_*z*_/d*z* of the normal component of the magnetic flux density *B*_*z*_ and the area sum *S* of the coil turns.

An EMEH with the coil resistance *R*_*C*_ delivers a voltage of

(10) $$\begin{array}{c} V_{L}\left(t\right)=\left(\frac{R_{L}}{R_{L}+R_{C}}\right)G\dot{z}\left(t\right) \end{array}$$

to the load resistance *R*_*L*_ connected to the device. Using Fourier analysis, the load voltage in the frequency domain

(11) $$\begin{array}{c} V_{L}\left(\omega \right)=\left(\frac{R_{L}}{R_{L}+R_{C}}\right)G\left|H\left(i\omega \right)\right|A\left(\omega \right) \end{array}$$

contains the complex frequency response

(12) $$\begin{array}{c} \left|H_{V}\left(i\omega \right)\right|=\left(\frac{R_{L}}{R_{L}+R_{C}}\right)G\left|H\left(i\omega \right)\right|. \end{array}$$

When the EMEH is subjected to a broad band random vibration, having *S*_*A*_, the SD of the base acceleration and the SD of the load voltage

(13) $$\begin{array}{c} S_{V_{L}}\left(\omega \right)={\left|H_{V}\left(i\omega \right)\right|}^{2}S_{A}\left(\omega \right) \end{array}$$

can be expressed in the parameters of the system as

(14) SVL(ω)=(RLRL+RC)2×G2(ω/ωn2)2[1−(ω/ωn)2]2+[2ζT(ω/ωn)]2SA(ω).

If the excitation is a stationary Gaussian random process with zero mean, the response of the system will also be a stationary Gaussian with a zero mean [25]. The mean square value of the load voltage

(15) VL2¯=∫−∞∞SVL(ω)dω=(RLRL+RC)2G2∫−∞∞|H(iω)|2SA(ω)dω

yields the average power delivered to the load resistance [24]

(16) $$\begin{array}{c} P_{L}=\frac{\bar{V_{L}^{2}}}{R_{L}}=\frac{1}{R_{L}}\int_{-\infty }^{\infty }S_{V_{L}}\left(\omega \right)d\omega =\int_{-\infty }^{\infty }S_{P_{L}}\left(\omega \right)\text{d}\omega . \end{array}$$

The SD of the power delivered to the load becomes

(17) SPL(ω)=1RLSVL(ω)=1RL(RLRL+RC)2 ×G2(ω/ωn2)2[1−(ω/ωn)2]2+[2ζT(ω/ωn)]2SA(ω).

#### 2.1.1. Broad Band White Noise Excitation

When the excitation is a stationary Gaussian white noise process, the SD of the acceleration, *S*_*A*_(*ω*), is flat and independent of frequency. Substituting the constant *S*_*A*_(*ω*) = *S*_0_ in (15) yields the mean square load voltage

(18) $$\begin{array}{c} \bar{V_{L}^{2}}=S_{0}{\left(\frac{R_{L}}{R_{L}+R_{C}}\right)}^{2}G^{2}\int_{-\infty }^{\infty }{\left|H\left(i\omega \right)\right|}^{2}d\omega . \end{array}$$

The integral in (18) is obtained by the method described in [26] that results in

(19) $$\begin{array}{c} \bar{V_{L}^{2}}=S_{0}{\left(\frac{R_{L}}{R_{L}+R_{C}}\right)}^{2}G^{2}\frac{\pi }{2\zeta_{T}\omega_{n}} \end{array}$$

and with (16) leads to the mean power delivered to the load

(20) $$\begin{array}{c} P_{L}=\frac{S_{0}}{R_{L}}{\left(\frac{R_{L}}{R_{L}+R_{C}}\right)}^{2}G^{2}\frac{\pi }{2\zeta_{T}\omega_{n}}. \end{array}$$

The total damping ratio *ζ*_*T*_ = *ζ*_*m*_ + *ζ*_*e*_ consists of the mechanical damping ratio

(21) $$\begin{array}{c} \zeta_{m}=\frac{1}{2Q_{m}} \end{array}$$

that is expressed in terms of the mechanical quality factor *Q*_*m*_ of the EMEH and the electrical damping ratio

(22) $$\begin{array}{c} \zeta_{e}=\frac{G^{2}}{2m\omega_{n}\left(R_{L}+R_{C}\right)} \end{array}$$

that can be obtained from the equivalent electrical circuit for the EMEH as described in [17].

By substituting for the total damping ratio *ζ*_*T*_, using (21) and (22), (20) becomes

(23) $$\begin{array}{c} P_{L}=m\pi S_{0}\frac{R_{L}}{R_{L}+R_{C}}\frac{G^{2}Q_{m}}{m\omega_{n}\left(R_{L}+R_{C}\right)+G^{2}Q_{m}}, \end{array}$$

which is more suitable to derive the optimum power condition for impedance matching. Optimizing (23) with respect to *R*_*L*_ yields the condition for optimum power transfer to the load as

(24) $$\begin{array}{c} R_{L,\text{opt}}=\sqrt{R_{C}^{2}+\frac{G^{2}Q_{m}R_{C}}{m\omega_{n}}}=\sqrt{R_{C}^{2}+\frac{G^{2}R_{C}}{b_{m}}}. \end{array}$$

Equation (24) reveals that the optimum load when an EMEH is subjected to random vibration is different from the optimum load *R*_*L*,opt_ = *R*_*C*_ + *G* ^2^/*b*_*m*_ when it is subjected to sinusoidal vibration [17].

The product *G* ^2^ *Q*_*m*_ in (23) is considered as the Product of merit (POM) for energy harvesters driven by random vibrations [24]. Increasing the product *G* ^2^ *Q*_*m*_ through modification of the design for an EMEH will increase the mean power delivered to the load. The mechanical quality factor *Q*_*m*_ can be increased by packaging the device in vacuum [27] or by incorporating air passages in the device design that allows air flow during operation to reduce damping [28]. For EMEHs with uniform magnetic field configuration the transformation factor *G* can be increased by increasing the magnetic flux density *B* and/or by increasing the effective length *L* of the coil within the constrained footprint of the device. For EMEHs with nonuniform magnetic field configuration, improving the transformation factor *G* requires increasing the gradient d*B*_*z*_/d*z* of the normal component of the magnetic flux density *B*_*z*_ and/or increasing the area sum *S* of the turns of the coil within the constrained footprint of the device. However, increasing the effective coil length or the number of coil turns also increases the coil resistance leading to higher electrical losses especially in MEMS scale EMEHs. Therefore, increasing the magnetic flux density *B* in uniform magnetic field configuration devices and the magnetic flux gradient in nonuniform magnetic field devices is a preferred method for increasing the transformation factor.

Values of the mechanical quality factor *Q*_*m*_ and of the transformation factor *G* for various MEMS scale EMEHs reported in the literature are summarized in Table 1. The mechanical quality factor for EMEHs ranges from 5.8 to 258.7. Due to their larger number of coil turns, wound coil type EMEHs exhibit higher values for the transformation factor that contribute to the higher values of the POM *G* ^2^ *Q*_*m*_ in comparison to planar coil type EMEHs. Since these values vary widely for different designs, they may not be suitable for comparing various devices. However, for a given device, the variation in its product of merit in response to its design parameter can be used to optimize its performance.

For simulation we used the dimensions and parameters (Table 2) of our EMEH described in [17], where the nonuniform magnetic field is caused by two permanent magnets with remanent flux density *B*_*r*_, which are suspended by a planar copper spring between two identical coils.

**Figure 2:**
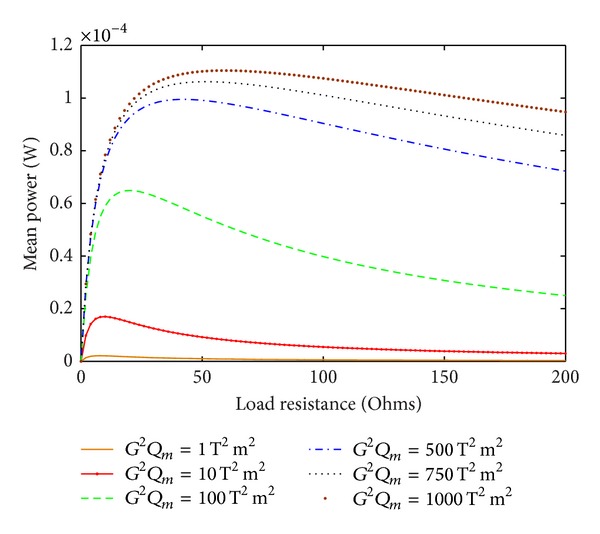
Mean power as a function of load resistance for different values of *G* ^2^ *Q*_*m*_.

**Figure 3:**
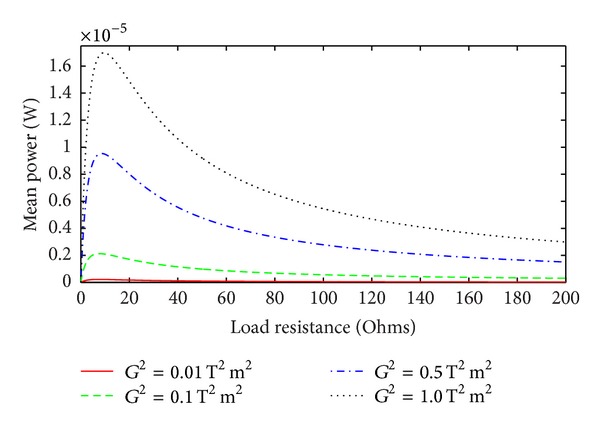
Mean power as a function of load resistance for different values of *G* ^2^ for *Q*_*m*_ = 5.7.

The mean power as a function of load resistance for various values of *G* ^2^ *Q*_*m*_ is shown in Figure 2. The computation was performed for the acceleration SD of *S*_*A*_(*ω*) = *S*_0_ = 0.01 g^2^/rad/s. The simulation results verify that there is an optimum value for the load resistance for each POM *G* ^2^ *Q*_*m*_ and that the optimum load resistance increases as the POM is increased. For higher values of the POM the curves become increasingly flat beyond the optimum load resistance. This indicates that the device will perform well even at the load resistance higher than the optimum.  Figure 3 shows the dependence of the mean power on the transformation factor as a function of load resistance. This corresponds to the situation where the mechanical quality factor *Q*_*m*_ for the EMEH is low and remains constant while the transformation factor varies. The curves in Figure 3 are more spiked in comparison to those in Figure 2, where the product *G* ^2^ *Q*_*m*_ is varied. This indicates that an EMEH becomes more sensitive to load resistance variations when its *Q*_*m*_ is low and where only the transformation factor is changed. Further, the optimum load resistance is quite different than for the case where the POM is increased.

From (14), (21), and (22) the SD of the load voltage for white noise base excitation becomes

(25) SVL(ω) =(RLRL+RC)2G2S0  ×((ωωn2)2×([1−(ωωn)2]2        +[(1Qm+G2(mωn(RL+RC)))          ×(ωωn)]2)−1).

**Figure 4:**
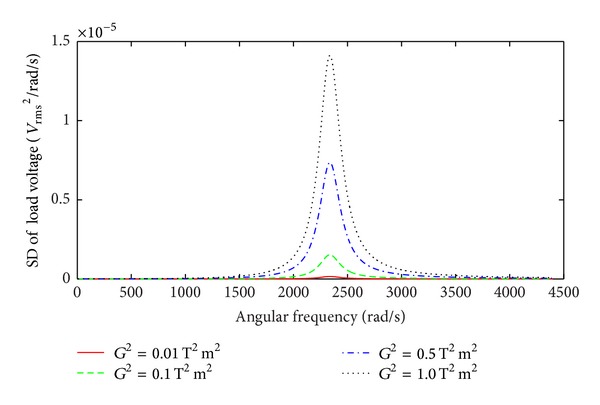
SD of the load voltage as a function of angular frequency for various values of *G* ^2^, *Q*_*m*_ = 5.7.

The SD of the load voltage as a function of angular frequency is shown in Figure 4 for different values of *G* ^2^. The SD of the load voltage shows a significant peak in the vicinity of the natural frequency of the linear EMEH. The EMEH acts as a mechanical filter and generates power in a limited band over the bandwidth

(26) $$\begin{array}{c} \Delta \omega =2\zeta_{T}\omega_{n}=2\left(\zeta_{m}+\zeta_{e}\right)\omega_{n}=\frac{\omega_{n}}{Q_{m}}+\frac{G^{2}}{m\left(R_{L}+R_{C}\right)}. \end{array}$$

A broader bandwidth for the EMEH is preferred in order to extract vibration energy from a wider band of random excitation. A higher transformation factor leads to wider bandwidth for the device. However, increasing the transformation factor by using a larger number of turns for the coil within a constrained area is undesirable, as this increases coil resistance that leads to power loss and negatively affects the bandwidth. As seen in Figure 2, it is more significant for the EMEH subjected to broad band vibration to optimize both *Q*_*m*_ and *G* ^2^; however, increasing the mechanical quality factor *Q*_*m*_ adversely affects the bandwidth of the device. This conflicting situation can be resolved by increasing the transformation factor through modifications to the magnetic flux density.

**Figure 5:**
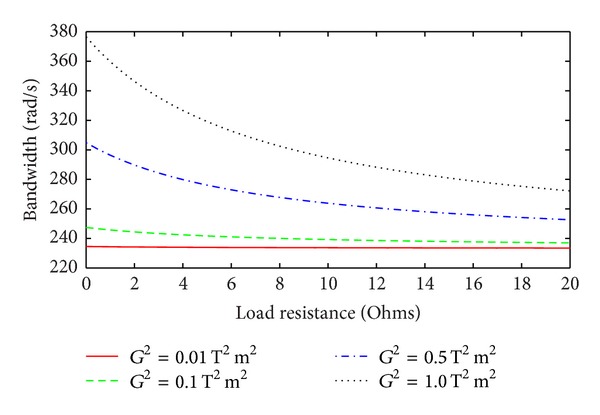
Linear EMEH bandwidth as a function of the load resistance for different values of *G* ^2^ and *Q*_*m*_ = 5.7.

Figure 5 shows the bandwidth of a linear EMEH as a function of the load resistance for several values of *G* ^2^ and *Q*_*m*_ = 5.7. Energy harvesters with a large transformation factor exhibit broader bandwidths that, however, drop sharply as the load resistance is increased. At a higher load resistance the contribution due to the transformation factor term in (26) is minimal and the device bandwidth is controlled by the dominant mechanical quality factor term. However, for EMEHs with a small transformation factor, the contribution due to the transformation factor term in (26) is negligible and the bandwidth becomes independent of the load resistance as evident in Figure 5.

The maximum value of the SD of the load voltage

(27) SVL1=SVL(ω=ωn)=RL2RL+RCmS0G2Qmωn(mωn(RL+RC)+G2Qm)

occurs at resonance and, likewise, the mean power also depends on the POM. Increasing the POM for the EMEH will lead to an increase in the peak value of the SD of the load voltage.

**Figure 6:**
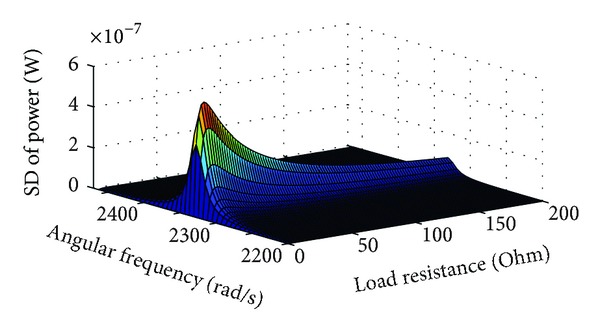
SD of the power as a function of angular frequency and load resistance for *Q*_*m*_ = 5.7 and *G* = 0.1 Tm.

The SD of the power as a function of angular frequency and load resistance from (21), (22), and (17) is shown in Figure 6. Similar to the SD of the load voltage it shows a narrow peak in the vicinity of the natural frequency and at the optimum load resistance.

#### 2.1.2. Band-Limited White Noise Excitation

When the linear EMEH is excited by a stationary band-limited Gaussian white noise *S*_*A*_(*ω*) = *S*_0_ between the angular frequency limits *ω*_1_ and *ω*_2_ the power spectral density of the load voltage is given by (25) for *ω*_1_ ≤ |*ω*| ≤ *ω*_2_ and is zero elsewhere.

**Figure 7:**
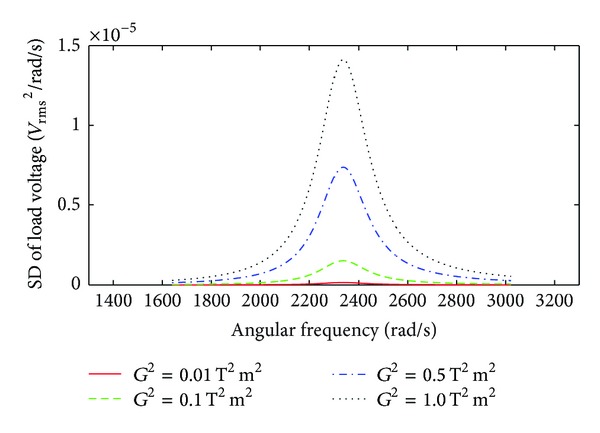
SD of the load voltage as a function of angular frequency for various values of *G* ^2^ at band-limited random excitation.

The SD of the load voltage for various values of *G* ^2^ for an EMEH excited by a band-limited random vibration from *ω*_1_ = 1640 rad/s to *ω*_2_ = 3022 rad/s is shown in Figure 7. The SD of the load voltage under band-limited excitation is maximum in the vicinity of the natural frequency similar to that of a broad band excitation, except that it is only nonzero over the frequency band of the input excitation.

Under band-limited Gaussian white noise random excitation, the mean square value of the load voltage

(28) $$\begin{array}{c} \bar{V_{L}^{2}}=S_{0}\left[\int_{-\omega_{2}}^{-\omega_{1}}{\left|H_{V}\left(i\omega \right)\right|}^{2}\text{d}\omega +\int_{\omega_{1}}^{\omega_{2}}{\left|H_{V}\left(i\omega \right)\right|}^{2}\text{d}\omega \right] \end{array}$$

when expressed in terms of the total damping ratio and the frequency ratio

(29) VL2¯=(RLRL+RC)2G2S0×[∫−ω2−ω1(ω/ωn2)2[1−(ω/ωn)2]2+[2ζT(ω/ωn)]2dω  +∫ω1ω2(ω/ωn2)2[1−(ω/ωn)2]2+[2ζT(ω/ωn)]2dω]

contains incomplete integrals that can be obtained by using the method of partial fraction expansion [26] or can be found with indefinite integral tables (e.g., by G. Petit Bois, 1961) [36]. Equation (29) can be written in a more compact form

(30) $$\begin{array}{c} \bar{V_{L}^{2}}={\left(\frac{R_{L}}{R_{L}+R_{C}}\right)}^{2}\frac{\pi G^{2}S_{0}}{2\zeta_{T}\omega_{n}}\left[\Gamma \left(\frac{\omega_{2}}{\omega_{n},\zeta_{T}}\right)-\Gamma \left(\frac{\omega_{1}}{\omega_{n},\zeta_{T}}\right)\right], \end{array}$$

where the integral factor Γ [36] can be expressed in terms of the frequency ratio and the total damping ratio as

(31) Γ(ωωn,ζT)=1πarc tan2ζT(ω/ωn)1−(ω/ωn)2−ζT2π1−ζT2×ln⁡1+(ω/ωn)2+21−ζT2(ω/ωn)1+(ω/ωn)2−21−ζT2(ω/ωn).

In (30) the terms in front of the bracket describe the mean square load voltage (variance) of the harvester due to broad band Gaussian white noise excitation in (19). The integral factor Γ in the brackets is the correction factor when the excitation is band-limited. For broad band Gaussian white noise excitation the value of the integral factor Γ(*∞*, *ζ*_*T*_) − Γ(0, *ζ*_*T*_) is 1, whereas for band-limited excitation it is always less than 1.

**Figure 8:**
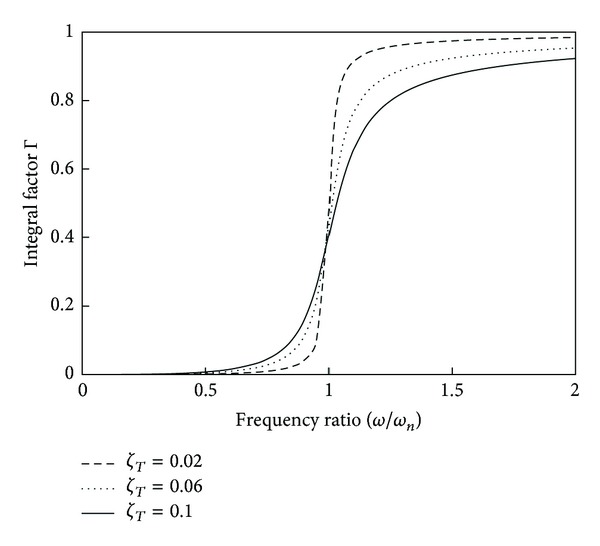
Integral factor for mean square load voltage of an EMEH subjected to band-limited Gaussian white noise.

For three values of the total damping ratio *ζ*_*T*_ = *ζ*_*m*_ + *ζ*_*e*_, the integral factor Γ is shown in Figure 8. The factor Γ increases monotonically as a function of the frequency ratio *ω*/*ω*_*n*_ with values residing between 0 and 1. Higher values of the mean square load voltage (or correction factor in the brackets) in (30) require lower values of the total damping factor. The electrical damping ratio *ζ*_*e*_ (or proportionally *G* ^2^) needs to be as high as possible for high power generation; therefore, for smaller values of the total damping ratio the mechanical quality factor should be increased and the associated reduction in the bandwidth of the device should be compensated by increasing the transformation factor.

## 3. Harvesters with Nonlinear Stiffness

For a nonlinear EMEH with linear damping force $d(\dot{z})=b_{T}\dot{z}$ and nonlinear spring force *s*(*z*) = *kz* + *ηkN*(*z*), the general equation of motion (1) of the harvester becomes

(32) $$\begin{array}{c} m\ddot{z}+b_{T}\dot{z}+\left[kz+\eta kN\left(z\right)\right]=-m\ddot{y}, \end{array}$$

in which the nonlinear spring force comprises of a linear stiffness component *kz* and a nonlinear stiffness component *kηN*(*z*). The scaling factor *η* and the nonlinear function *N*(*z*) represent the nonlinearity of the stiffness of the harvester. For an EMEH with a symmetric suspension spring, the potential energy is a symmetric (even) function of *z* and that requires the spring force to be an antisymmetric polynomial (odd function) of *z*. The nonlinear function *N*(*z*) is therefore a polynomial with only odd terms. The scaling factor *η* represents the magnitude of the nonlinearity of the spring.

A nonlinear spring force that is common in EMEHs with a polymeric membrane as the restoring member [28] can be modeled to good approximation by a Duffing spring with the nonlinear spring force *s*(*z*) = *kz* + *kηz* ^3^. By substituting for *s*(*z*) and expressing in terms of the linear natural frequency *ω*_*n*_ and the total damping ratio *ζ*_*T*_, (32) results in

(33) $$\begin{array}{c} \ddot{z}+2\zeta_{T}\omega_{n}\dot{z}+\omega_{n}^{2}\left(z+\eta z^{3}\right)=-\ddot{y}. \end{array}$$

For a stationary Gaussian random excitation with zero mean, the response of the harvester will also be stationary Gaussian with zero mean. The solution of (33) can be obtained by the method of statistical linearization [37–40]. The replacement of the nonlinear component *ω*_*n*_^2^(*z* + *ηz* ^3^) by an equivalent linear component *ω*_eq_^2^ *z* yields the equation of motion of an equivalent linear energy harvester

(34) $$\begin{array}{c} \ddot{z}+2\zeta_{\text{e}{\text{q}}_{T}}\omega_{\text{eq}}\dot{z}+\omega_{\text{eq}}^{2}z=-\ddot{y}, \end{array}$$

that depends on the equivalent damping ratio *ζ*_eq_*T*__ = (*ω*_*n*_/*ω*_eq_)*ζ*_*T*_ and the equivalent frequency *ω*_eq_ of an equivalent linear EMEH. To obtain an approximate solution for the response of the nonlinear harvester, the mean square value, *E*[*e* ^2^], of the error

(35) $$\begin{array}{c} e=\omega_{n}^{2}\left(z+\eta z^{3}\right)-\omega_{\text{eq}}^{2}z \end{array}$$

which would be produced by representing the nonlinear harvester by an equivalent linear harvester, must be minimized for the square of the equivalent frequency *ω*_eq_; that is, the equation

(36) $$\begin{array}{c} \frac{d}{d\omega_{\text{eq}}^{2}}E\left[e^{2}\right]=0 \end{array}$$

must be satisfied.

Substituting (35) into (36) and differentiating the resulting equation yields the expression for the equivalent frequency

(37) $$\begin{array}{c} \omega_{\text{eq}}^{2}=\omega_{n}^{2}\frac{E\left[z.z\left(1+\eta z^{2}\right)\right]}{\sigma_{z}^{2}} \end{array}$$

in terms of the standard deviation *σ*_*z*_ of the relative displacement *z*(*t*). Using the method described in [37] reduces (37) to a much simpler form

(38) $$\begin{array}{c} \omega_{\text{eq}}^{2}=\omega_{n}^{2}\left(1+3\eta \sigma_{z}^{2}\right). \end{array}$$

For a Gaussian white noise random excitation, the variance of the relative displacement

(39) $$\begin{array}{c} \sigma_{z}^{2}=\int_{-\infty }^{\infty }S_{z}\left(\omega \right)d\omega =S_{0}\int_{-\infty }^{\infty }{\left|H\left(i\omega \right)\right|}^{2}d\omega \end{array}$$

can be solved for the complex frequency response

(40) $$\begin{array}{c} H\left(i\omega \right)=\frac{-1}{-\omega^{2}+i2\zeta_{{\text{eq}}_{T}}\omega_{\text{eq}}\omega +\omega_{\text{eq}}^{2}} \end{array}$$

with the method described in [26, 37] that results in

(41) $$\begin{array}{c} \sigma_{z}^{2}=\frac{\pi S_{0}}{2\zeta_{{\text{eq}}_{T}}\omega_{\text{eq}}^{3}}=\frac{\pi S_{0}}{2\zeta_{T}\omega_{n}\omega_{\text{eq}}^{2}}. \end{array}$$

Using (38) the elimination of the equivalent frequency *ω*_eq_ from (41) yields a quadratic equation in *σ*_*z*_^2^

(42) $$\begin{array}{c} 3\eta \sigma_{z}^{4}+\sigma_{z}^{2}=\sigma_{z_{\text{Lin}}}^{2}=\frac{\pi S_{0}}{2\zeta_{T}\omega_{n}^{3}} \end{array}$$

where *σ*_*z*_Lin__ is the standard deviation of the relative displacement for the linear case where *η* = 0.

By substituting the positive root

(43) $$\begin{array}{c} \sigma_{z}^{2}=\frac{\sqrt{1+12\eta \sigma_{z_{\text{Lin}}}^{2}}-1}{6\eta } \end{array}$$

of (42) in (38) we obtain the equivalent frequency *ω*_eq_

(44) $$\begin{array}{c} \omega_{\text{eq}}^{2}=\omega_{n}^{2}\left[1+\frac{\sqrt{1+12\eta \sigma_{z_{\text{Lin}}}^{2}}-1}{2}\right], \end{array}$$

that minimizes the error *e*.

Equations (10) and (34) yield the equation

(45) 1G(RL+RCRL)[ddtVL(t)+2ζeqTωeqVL(t)+ωeq2∫VL(t)dt]  =−y¨(t),

which by Fourier analysis results in the frequency response of the harvester

(46) HV(iω)=(R  L  RL+RC)Gω(ωeq2−ω2)i−2ζeqTωeqω.

A Gaussian white noise base excitation *S*_*A*_(*ω*) = *S*_0_ yields the SD of the load voltage

(47) SVL(ω)=(RLRL+RC)2 ×G2S0(ω/ωeq2)2[1−(ω/ωeq)2]2+[2ζeqT(ω/ωeq)]2=(RLRL+RC)2G2S0 ×((ωωeq2)2   ×([1−(ωωeq)2]2     +[(ωnωeq)(1Qm+G2(mωn(RL+RC)))     ×(ωωeq)]2)−1).

Similar to (19), the mean square load voltage of the harvester

(48) $$\begin{array}{c} \bar{V_{L}^{2}}=S_{0}{\left(\frac{R_{L}}{R_{L}+R_{C}}\right)}^{2}G^{2}\frac{\pi }{2\zeta_{{\text{eq}}_{T}}\omega_{\text{eq}}} \end{array}$$

and the mean power delivered to the load

(49) $$\begin{array}{c} P_{L}=\frac{S_{0}}{R_{L}}{\left(\frac{R_{L}}{R_{L}+R_{C}}\right)}^{2}G^{2}\frac{\pi }{2\zeta_{{\text{eq}}_{T}}\omega_{\text{eq}}} \end{array}$$

can be derived here as functions of the equivalent total damping ratio and the equivalent frequency.

The parameters (Table 3) of our nonlinear membrane type EMEH [28] are used as reference values for simulating nonlinear EMEHs. The EMEH has a nonuniform magnetic field caused by two permanent magnets, with remanent flux density *B*_*r*_, that are suspended by a polydimethylsiloxane (PDMS) membrane between two identical coils.

**Figure 9:**
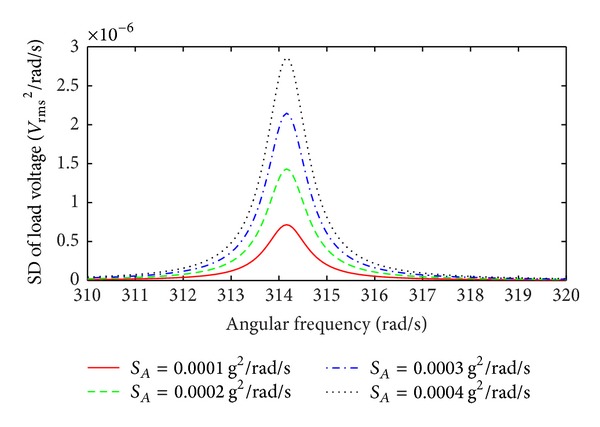
SD of the load voltage as a function of angular frequency for low levels of broad band Gaussian white noise random excitation; scaling factor *η* = 5 m^−2^.

The SD of the load voltage for a 100 Ω load at low levels of broad band Gaussian white noise random vibration is shown in Figure 9. The simulation is the result of (44) and (47) for a scaling factor *η* = 5 m^−2^ and a mechanical quality factor *Q*_*m*_ = 300. Under low levels of random vibrations the contribution of the second term in (44) is negligible. As a result, the resonant frequency is stable (not changing with increased base excitation), showing a linear response of the device. Therefore, the nonlinear EMEH operates in the linear regime under low levels of broad band random vibrations, where the relative displacement of the magnets is too small to cause a significant contribution from the nonlinear spring stiffness term.

**Figure 10:**
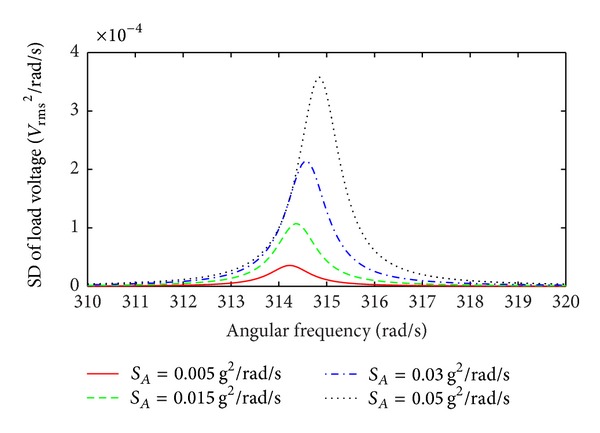
SD of the load voltage as a function of angular frequency for high levels of broad band Gaussian white noise random excitation; scaling factor *η* = 5 m^−2^.

The simulation results of the SD of the load voltage for higher levels of broad band Gaussian white noise random vibrations are shown in Figure 10. The maximum value of the load voltage spectrum increases with increasing base acceleration; moreover, the central frequency of the load voltage SD shifts towards higher frequencies and this is attributed to the increase in the spring stiffness (resonant frequency) of the device when it is subjected to stronger levels of random excitation. At relatively high base acceleration the large relative displacement of the mass invokes the nonlinear spring stiffening term, and the EMEH then operates in the nonlinear regime where the resonant frequency *ω*_eq_ given by (44) increases with increasing base acceleration. Moreover, in comparison to the load voltage SD under low levels of acceleration (Figure 9) at high levels of base acceleration the SD of the load voltage slightly broadens, increasing the bandwidth of the device.

## 4. Harvester with Nonlinear Stiffness and Nonlinear Damping

For EMEH with nonlinear damping $d(\dot{z})=b_{T}\dot{z}+b_{T}\alpha D(\dot{z})$ and nonlinear stiffness *s*(*z*) = *kz* + *kηN*(*z*), the general form of the equation of motion (1) of the harvester becomes

(50) $$\begin{array}{c} m\ddot{z}+\left[b_{T}\dot{z}+b_{T}\alpha D\left(\dot{z}\right)\right]+\left[kz+k\eta N\left(z\right)\right]=-m\ddot{y}. \end{array}$$

A good approximation to this is obtained by assuming the nonlinear EMEH as a Duffing oscillator, with linear-plus-cubic damping. The equation of motion for such a nonlinear EMEH

(51) $$\begin{array}{c} \ddot{z}+2\zeta_{T}\omega_{n}\left(\dot{z}+\alpha {\dot{z}}^{3}\right)+\omega_{n}^{2}\left(z+\eta z^{3}\right)=-\ddot{y} \end{array}$$

contains the nonlinear damping force $d(\dot{z})=b_{T}\dot{z}+b_{T}\alpha {\dot{z}}^{3}$ that consists of a linear damping component $b_{T}\dot{z}$ and the nonlinear damping component $b_{T}\alpha {\dot{z}}^{3}$, with *α* as the scaling factor.

When the excitation and response of the harvester are both stationary Gaussian with zero mean, the solution of (51) can also be obtained by the method of statistical linearization [37–40]. The replacement of the nonlinear damping force $2\zeta_{T}\omega_{n}(\dot{z}+\alpha {\dot{z}}^{3})$ and the nonlinear spring force *ω*_*n*_^2^(*z* + *ηz* ^3^) by an equivalent linear damping force $\mu_{\text{eq}}\dot{z}=2\zeta_{{\text{eq}}_{T}}\omega_{\text{eq}}\dot{z}$ and equivalent linear spring force *ω*_eq_^2^ *z*, respectively, yields the equation of motion of an equivalent linear energy harvester

(52) $$\begin{array}{c} \ddot{z}+\mu_{\text{eq}}\dot{z}+\omega_{\text{eq}}^{2}z=-\ddot{y}. \end{array}$$

To obtain an approximate solution for the response of the nonlinear harvester, the error

(53) $$\begin{array}{c} e=2\zeta_{T}\omega_{n}\left(\dot{z}+\alpha {\dot{z}}^{3}\right)+\omega_{n}^{2}\left(z+\eta z^{3}\right)-\mu_{\text{eq}}\dot{z}-\omega_{\text{eq}}^{2}z \end{array}$$

resulting from this assumption must be minimized. The mean square of the error *E*[*e* ^2^] is to be minimized with respect to square of the equivalent frequency *ω*_eq_ and equivalent damping coefficient term *μ*_eq_; that is, equations

(54) $$\begin{array}{c} \frac{\partial }{\partial \omega_{\text{eq}}^{2}}E\left[e^{2}\right]=0,\\ \frac{\partial }{\partial \mu_{\text{eq}}}E\left[e^{2}\right]=0 \end{array}$$

must be satisfied. By substituting (53) into (54), two simultaneous equations

(55) E[z˙2ζTωn(z˙+αz˙3)+ωn2(z+ηz3)]  −μeqE[z˙2]−ωeq2E[zz˙]=0,E[z2ζTωn(z˙+αz˙3)+ωn2(z+ηz3)]  −μeqE[zz˙]−ωeq2E[z2]=0,

are obtained for the equivalent damping term *μ*_eq_ and the equivalent frequency *ω*_eq_.

For the relative displacement *z*(*t*) being a stationary Gaussian random process, with zero mean, the substitutions $E[z\dot{z}]=0$, *E*[*z* ^2^] = *σ*_*z*_^2^, and $E[{\dot{z}}^{2}]=\sigma_{\dot{z}}^{2}$ [37–39] in (55) yield the relation for the equivalent damping term

(56) $$\begin{array}{c} \mu_{\text{eq}}=\frac{E\left[\dot{z}2\zeta_{T}\omega_{n}\left(\dot{z}+\alpha {\dot{z}}^{3}\right)+\omega_{n}^{2}\left(z+\eta z^{3}\right)\right]}{\sigma_{\dot{z}}^{2}}, \end{array}$$

as a function of the standard deviation $\sigma_{\dot{z}}$ of the relative velocity $\dot{z}(t)$ as well as the relation for the equivalent frequency

(57) $$\begin{array}{c} \omega_{\text{eq}}^{2}=\frac{E\left[z2\zeta_{T}\omega_{n}\left(\dot{z}+\alpha {\dot{z}}^{3}\right)+\omega_{n}^{2}\left(z+\eta z^{3}\right)\right]}{\sigma_{z}^{2}}, \end{array}$$

as a function of the standard deviation *σ*_*z*_ of the relative displacement *z*(*t*). The assumption of *z*(*t*) and $\dot{z}(t)$ being both Gaussian yields the much simpler equations

(58) $$\begin{array}{c} \mu_{\text{eq}}=E\left[\frac{\partial }{\partial \dot{z}}\left[2\zeta_{T}\omega_{n}\left(\dot{z}+\alpha {\dot{z}}^{3}\right)+\omega_{n}^{2}\left(z+\eta z^{3}\right)\right]\right],\\ \omega_{\text{eq}}^{2}=E\left[\frac{\partial }{\partial z}\left[2\zeta_{T}\omega_{n}\left(\dot{z}+\alpha {\dot{z}}^{3}\right)+\omega_{n}^{2}\left(z+\eta z^{3}\right)\right]\right], \end{array}$$

which after differentiation result in the equivalent damping term

(59) $$\begin{array}{c} \mu_{\text{eq}}=E\left[2\zeta_{T}\omega_{n}\left(1+3\alpha {\dot{z}}^{2}\right)\right]=\mu_{T}\left(1+3\alpha \sigma_{\dot{z}}^{2}\right), \end{array}$$

as a function of the linear damping term *μ*_*T*_ = 2*ζ*_*T*_ *ω*_*n*_ of the linear EMEH where *α* = 0 and the equivalent frequency

(60) $$\begin{array}{c} \omega_{\text{eq}}^{2}=E\left[\omega_{n}^{2}\left(1+3\eta z^{2}\right)\right]=\omega_{n}^{2}\left(1+3\eta \sigma_{z}^{2}\right) \end{array}$$

in terms of the natural frequency *ω*_*n*_ of the linear case with *η* = 0.

For a Gaussian white noise random excitation, the variance of the relative displacement of the equivalent linear EMEH

(61) $$\begin{array}{c} \sigma_{z}^{2}=\frac{\pi S_{0}}{2\zeta_{{\text{eq}}_{T}}\omega_{\text{eq}}^{3}}=\frac{\pi S_{0}}{\mu_{\text{eq}}\omega_{\text{eq}}^{2}} \end{array}$$

and the variance of the relative velocity

(62) $$\begin{array}{c} \sigma_{\dot{z}}^{2}=\frac{\pi S_{0}}{2\zeta_{{\text{eq}}_{T}}\omega_{\text{eq}}}=\frac{\pi S_{0}}{\mu_{\text{eq}}} \end{array}$$

can be determined with the method described in [26, 37] as before.

With the variance of the relative velocity of the linear EMEH

(63) $$\begin{array}{c} \sigma_{{\dot{z}}_{L}}^{2}=\frac{\pi S_{0}}{2\zeta_{{\;}_{T}}\omega_{n}}=\frac{\pi S_{0}}{\mu_{T}} \end{array}$$

and (62), elimination of *μ*_eq_ and *μ*_*T*_ from (63) yields a quadratic equation for the variance of the relative velocity $\sigma_{\dot{z}}^{2}$

(64) $$\begin{array}{c} \sigma_{\dot{z}}^{2}\left(1+3\alpha \sigma_{\dot{z}}^{2}\right)=\sigma_{{\dot{z}}_{L}}^{2}. \end{array}$$

Substitution of the positive root

(65) $$\begin{array}{c} \frac{\sigma_{\dot{z}}^{2}}{\sigma_{{\dot{z}}_{L}}^{2}}=\frac{\mu_{T}}{\mu_{\text{eq}}}=\frac{\sqrt{1+12\alpha \sigma_{{\dot{z}}_{L}}^{2}}-1}{6\alpha \sigma_{{\dot{z}}_{L}}^{2}}, \end{array}$$

of (64) into (59) yields the relation for the equivalent damping term:

(66) $$\begin{array}{c} \mu_{\text{eq}}=\mu_{T}\left[1+\frac{\sqrt{1+12\alpha \sigma_{{\dot{z}}_{L}}^{2}}-1}{2}\right]. \end{array}$$

Similarly with the variance of the relative displacement of the linear EMEH

(67) $$\begin{array}{c} \sigma_{z_{L}}^{2}=\frac{\pi S_{0}}{2\zeta_{T}\omega_{n}^{3}}=\frac{\pi S_{0}}{\mu_{T}\omega_{n}^{2}} \end{array}$$

and (61), elimination of *ω*_eq_ and *ω*_*n*_ from (60) yields a quadratic equation for the variance of the relative displacement *σ*_*z*_^2^:

(68) $$\begin{array}{c} \sigma_{z}^{2}\left(1+3\eta \sigma_{z}^{2}\right)\frac{\mu_{\text{eq}}}{\mu_{T}}=\sigma_{z_{L}}^{2}. \end{array}$$

Substitution of the positive root

(69) $$\begin{array}{c} \frac{\sigma_{z}^{2}}{\sigma_{z_{L}}^{2}}=\left(\frac{\sqrt{1+12L}-1}{6L}\right)\frac{\mu_{T}}{\mu_{\text{eq}}} \end{array}$$

of (68) into (60) yields the relation for the equivalent natural frequency

(70) $$\begin{array}{c} \omega_{\text{eq}}^{2}=\omega_{n}^{2}\left[1+\frac{\sqrt{1+12L}-1}{2}\right], \end{array}$$

where

(71) $$\begin{array}{c} L=\eta \sigma_{z_{L}}^{2}\frac{\mu_{T}}{\mu_{\text{eq}}}=\eta \sigma_{z_{L}}^{2}{\left[1+\frac{\sqrt{1+12\alpha \sigma_{{\dot{z}}_{L}}^{2}}-1}{2}\right]}^{-1}. \end{array}$$

For a Gaussian white noise base excitation *S*_*A*_(*ω*) = *S*_0_, the SD of the load voltage

(72) SVL(ω)=(RLRL+RC)2G2S0×(ω/ωeq2)2[1−(ω/ωeq)2]2+[2ζeqT(ω/ωeq)]2

for EMEHs with combined stiffness and damping nonlinearities can be obtained by a similar procedure described in Section 3 for EMEHs with only nonlinear stiffness.

Substituting *μ*_eq_ = 2*ζ*_eq_*T*__ *ω*_eq_ and *μ*_*T*_ = 2*ζ*_*T*_ *ω*_*n*_ in (66) yields the equivalent total damping ratio

(73) ζeqT=ωnζTωeq[1+1+12ασz˙L2−12]=ωnωeq[1Qm+G2mωn(RL+RC)] ×[1+12(1+12ασz˙L2−1)]=ωn2ωeq[1Qm+G2mωn(RL+RC)] ×[1+12(1+12παmS0Qm(RL+RC)mωn(RL+RC)+G2Qm−1)].

Similarly, (71) becomes

(74) L=ηπmS0Qm(RL+RC)ωn2[mωn(RL+RC)+G2Qm]×[1+12(1+12παmS0Qm(RL+RC)mωn(RL+RC)+G2Qm−1)]−1.

These allow computing the mean square load voltage

(75) $$\begin{array}{c} \bar{V_{L}^{2}}=S_{0}{\left(\frac{R_{L}}{R_{L}+R_{C}}\right)}^{2}G^{2}\frac{\pi }{2\zeta_{\text{eq}}\omega_{\text{eq}}} \end{array}$$

and the mean power

(76) $$\begin{array}{c} P_{L}=\frac{S_{0}}{R_{L}}{\left(\frac{R_{L}}{R_{L}+R_{C}}\right)}^{2}G^{2}\frac{\pi }{2\zeta_{\text{eq}}\omega_{\text{eq}}} \end{array}$$

delivered to the load.

**Figure 11:**
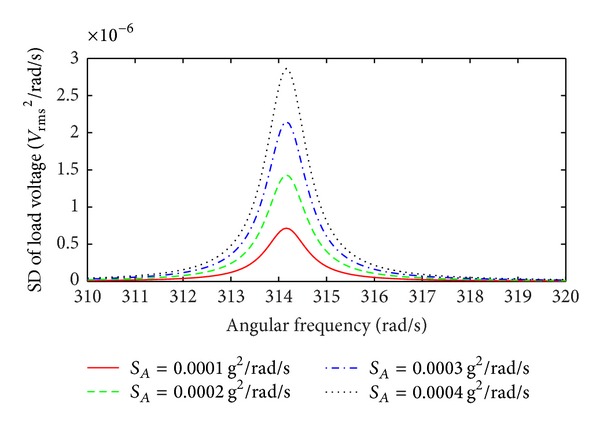
SD of the load voltage as a function of angular frequency for low levels of broad band Gaussian white random excitation; spring scaling factor *η* = 5 m^−2^ and damping scaling factor *α* = 0.05 s^2^ m^−2^.

Figure 11 shows the SD of the load voltage of an EMEH with nonlinear damping and nonlinear stiffness, for a 100 Ω load, at low levels of broad band Gaussian white random vibration. The simulation results are based on (70), (72), and (73) with a spring scaling factor *η* = 5 m^−2^ and a damping scaling factor *α* = 0.05 s^2^ m^−2^. In comparison to the load voltage SD of an EMEH with linear damping and nonlinear stiffness in Figure 9, almost the same response is obtained. Under such low levels of base acceleration the linear damping and linear stiffness terms are dominant, whereas the nonlinear damping and nonlinear stiffness terms have negligible contributions due to the small values of the standard deviation of the relative velocity and the relative displacement, respectively. At low base accelerations the second term in (70) and (73) is negligible, so that the nonlinear EMEH operates in the linear regime with a stable central frequency (resonant frequency) of the load voltage SD.

**Figure 12:**
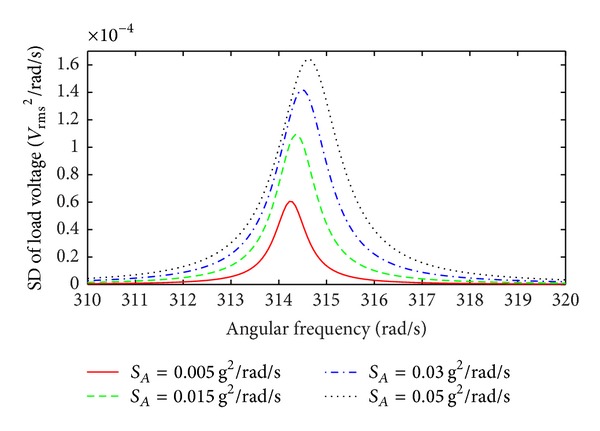
SD of the load voltage as a function of angular frequency for high levels of broad band Gaussian white noise random excitation; spring scaling factor *η* = 5 m^−2^ and damping scaling factor *α* = 0.05 s^2^ m^−2^.

The simulation results of EMEHs with nonlinear damping and stiffness at relatively high levels of broad band Gaussian white noise random vibrations are shown in Figure 12. With an increased base acceleration level, the shift of the central frequency of the load voltage SD towards higher frequencies indicates the operation of the device in the nonlinear regime. Under these conditions, the higher values of the standard deviations of the relative velocity and the relative displacement of the mass invoke the nonlinear effects of the system. In other words, the contribution of the second terms in (70) and (73) becomes significant.

In comparison to the response of EMEHs with nonlinear stiffness only as in Figure 10, the same shift in the SD maximum value and central frequency is evident in the case of the fully nonlinear harvester. However, in Figure 12, these shifts are less significant due to the existence of the nonlinear damping. The nonlinear damping of the EMEH, which increases as the standard deviation of the relative velocity rises, does not allow the same increase in the maximum value for the load voltage SD and the central frequency as in case of the EMEH with linear damping. Moreover, the higher damping leads to broader bandwidths in comparison to the EMEH with nonlinear stiffness only in Figure 10.

**Figure 13:**
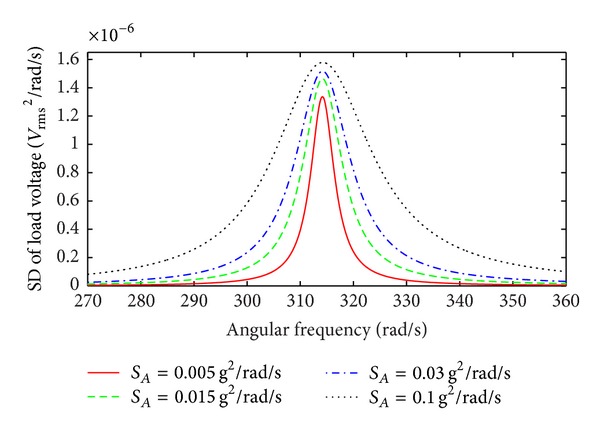
SD of the load voltage as a function of angular frequency for high levels of broad band Gaussian white random excitation; spring scaling factor *η* = 5 m^−2^ and damping scaling factor *α* = 5 s^2^ m^−2^.

The response of the nonlinear EMEH with a larger damping scaling factor *α* = 5 s^2^ m^−2^ is shown in Figure 13. In this case a much smaller increase in the maximum value of the load SD is seen; moreover, the central frequency is almost constant and does not change with increasing base acceleration. Broader bandwidths are obtained in comparison to a nonlinear EMEH with smaller damping scaling factor *α* = 0.05 s^2^ m^−2^. The larger value of the nonlinear damping term diminishes the effects of the nonlinear stiffness term, until and unless the spring scaling factor is very large.

**Figure 14:**
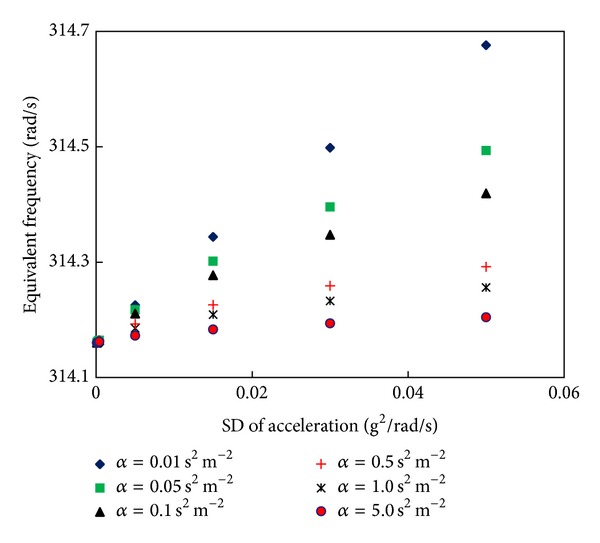
Equivalent frequency as a function of SD of acceleration for several values of the damping scaling factor *α* with *η* = 5 m^−2^.

For a nonlinear EMEH with combined nonlinear stiffness and damping, the equivalent resonant frequency for a stiffness damping factor of *η* = 5 m^−2^ and several values for the damping scaling factor *α* is plotted in Figure 14. When the EMEH is subjected to increasing SD levels of the acceleration, for smaller values of *α*, the shift in the equivalent frequency of the response is significant; however, this shift decreases as *α* is increased. For *α* = 5 s^2^ m^−2^ or larger values, the change in the equivalent frequency is negligible. Moreover, it can be seen from the plot that at lower SD levels of the acceleration (e.g., at *S*_*A*_ = 0.0001 g^2^/rad/s) the shift in resonant frequency is minimal even if the difference in *α* is large. This indicates that at the excitation level equal to or less than 0.0001 g^2^/rad/s, contributions from the nonlinear effects are negligible. The EMEH will be operating in the linear regime with approximately constant resonant (central) frequency of the SD of the response.

**Figure 15:**
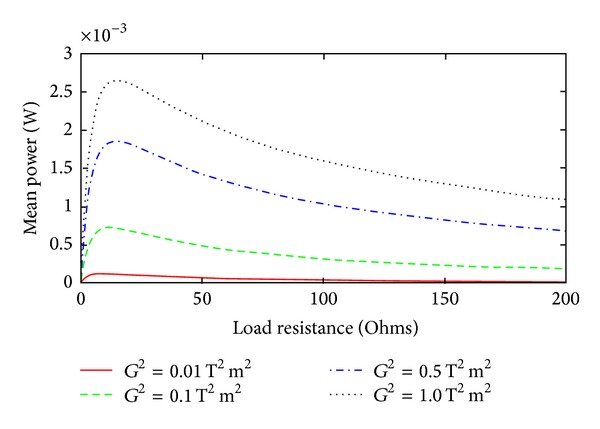
Mean power as a function of load resistance for different values of *G* ^2^ with spring scaling factor *η* = 5 m^−2^ and the damping scaling factor *α* = 5 s^2^ m^−2^ at SD = 0.01 g^2^/rad/s.

The mean power as a function of load resistance for several values of the transformation factor is shown in Figure 15. The plots are obtained by using (70) and (73) in (76). The computation is performed for the acceleration SD of 0.01 g^2^/rad/s. With increase in the transformation factor, the peak value of the mean power increases and the optimum load changes.

## 5. Conclusions

Analytical models for linear and nonlinear electromagnetic energy harvesters (EMEHs) for random vibrations have been developed and simulations were performed to predict the behaviour of these harvesters under broad band random excitations. In contrast to harmonic excitation, the simulation results have shown different output responses when linear and nonlinear EMEHs are subjected to broad band random excitation.

For linear EMEHs under broad band stationary Gaussian random excitation, the simulation results of the mean power, mean square load voltage, spectral density of the device output, and the harvester bandwidth show the significance of both the transformation and the mechanical quality factors. For larger values of the product of merit the mean power becomes less dependent on the optimum load and the device can be operated off its optimum load condition. The increase of the transformation factor for EMEHs with a small mechanical quality factor required the device to be operated at the optimum load for better performance. Moreover, the optimum load condition under random vibration is quite different from that of harmonic excitations. Higher values of the transformation factor have shown to broaden the bandwidth of the harvester and the bandwidth is dependent on the load resistance in such a case; however, at lower transformation factor, the bandwidth solely depends on the mechanical quality factor and is independent of the load resistance.

For linear EMEHs the SD of the load voltage under band-limited random excitation is nonzero only over the input frequency band and is maximum in the vicinity of the natural frequency similar to that of broad band excitation. The mean square load voltage depends on the frequency band of the excitation and the total damping ratio of the harvester. Higher values of the mean square of the load voltage require small values of the total damping ratio. The electrical damping ratio (transformation factor) should be as high as possible for high power generation, therefore for smaller values of the total damping ratio the mechanical quality factor must be increased. The associated reduction in the bandwidth of the harvester must then be compensated by increasing the transformation factor.

For nonlinear EMEHs the statistical linearization method was used for modeling under broad band random vibration. The response of nonlinear EMEHs not only depends on the spectral density of the base acceleration but also on the standard deviations of the relative velocity and the relative displacement. Under low levels of random excitation, the contribution from the nonlinear terms is negligible, the linear stiffness and linear damping are dominant, harvesters operate in the linear regime, and the response of a nonlinear device is just similar to a linear EMEH. When these nonlinear harvesters are subjected to higher levels of random excitations to invoke the nonlinear effects, the simulation results have shown that not only the maximum value of the load voltage is increased but also the central (resonant) frequency of the spectral density has been shifted towards higher frequencies. The shift in the central frequency is attributed to the increased stiffness. However, this shift becomes less significant in case of EMEHs with nonlinear damping, since the nonlinear damping term contributes inversely to the resonance frequency. Moreover, slightly broader bandwidths are obtained in the nonlinear regime in comparison to operating in the linear regime. The presence of high levels of nonlinear damping not only increases the bandwidth of the harvester at the expense of decreased peak value of the load voltage spectral density, but it also leads to a stable resonant frequency even at relatively high levels of random excitation.

## Figures and Tables

**Table 1:** Parameters of MEMS scale electromagnetic energy harvesters.

Coil type	*m* (kg)	*F*_resonant_ (Hz)	*R*_*C*_ (Ω)	*R*_*L*_ (Ω)	Mech. quality factor *Q*_*m*_	Elect. quality factor *Q*_*e*_	Total quality factor *Q*_*T*_	*G* (Tm)	*G* ^2^ *Q*_*m*_ (T^2^m^2^)	References
Wound	42.8 × 10^−3^	13.11	18		117.52	6.53^a^		3.118^b^	1142.52	[29]
	25 × 10^−3^	84	3.65			329.88^a^		0.3821^b^		[29]
	3.8 × 10^−3^	94.8	1.2	2.7	258.7^c^	24.61^a^		0.3322^b^	28.55	[30]
	0.44 × 10^−3^	350	93	100	216	1120	181	0.41^b^	36.31	[31]
	0.028 × 10^−3^	9.5 k		100			164			[31]
		52.1	100	200	232	243	119			[6]
	1.02 × 10^−3^	50	2323			210–60		6.04		[32]
			1530					4.43		[32]
	0.028 × 10^−3^	8.08 k	112		26					[33]
		208			121.8					[34]
		106			141.3					[34]

Planar	4.036 × 10^−3^	24.8	100	100	23.36					[19]
	0.0304 × 10^−3^	100	2	4	16.2^c^	403.23^a^	7.94^c^	0.017^ b^	0.005	[35]
	0.014 × 10^−3^	9.837 k	55		164					[33]
	0.54 × 10^−3^	60	110	110	48.5^c^	2345.33^a^		0.0691^b^	0.232	[33]
			31	39	136		120	1.5 × 10^−3^	0.0003	[27]
					221^d^		207^d^			[27]
	0.93 × 10^−3^	371	7.5	100	5.7		5.83^c^	0.075	0.033	[17]

^a^Calculated using equation *Q*_*e*_ = 1/2ζ_*e*_.

^b^Calculated using equation $G=\sqrt{\left(R_{L}+R_{C})(2\pi mF/Q_{e}\right)}$.

^c^Calculated using equation *Q*_*m*_ = 1/2ζ_*m*_ = 2π*mF*/*b*_*m*_.

^d^Determined from testing in vacuum.

**Table 2:** Dimensions and parameters of the EMEH prototype [17].

Description	Value
Device size	12 mm × 12 mm × 7 mm
Magnet (NdFeB)	1.3 T
Mass of each magnet	0.465 g
Square spiral coil envelop	8 mm × 8 mm
Resistance of coil *R*_*C*_	7.5 Ω
Mechanical quality factor *Q*_*m*_	5.7
Resonant frequency *F*_resonant_	371 Hz
Transformation factor *G*	0.075 Tm

**Table 3:** Dimensions and parameters of the nonlinear EMEH prototype [28].

Description	Value
Device size	15 mm × 15 mm × 10 mm
Magnet (NdFeB)	1.32 T
Mass of each magnet	0.93 g
Square spiral coil envelop	8 mm × 8 mm
Resistance of coil	10.1 Ω
Linear resonant frequency	50 Hz
